# Morphological and Molecular Phylogenetic Data Reveal a New Species of *Primula* (Primulaceae) from Hunan, China

**DOI:** 10.1371/journal.pone.0161172

**Published:** 2016-08-31

**Authors:** Yuan Xu, Xun-Lin Yu, Chi-Ming Hu, Gang Hao

**Affiliations:** 1 Key Laboratory of Plant Resources Conservation and Sustainable Utilization, South China Botanical Garden, the Chinese Academy of Sciences, Guangzhou, China; 2 College of Forestry, Central South University of Forestry and Technology, Changsha, China; 3 College of Life Sciences, South China Agricultural University, Guangzhou, China; The National Orchid Conservation Center of China; The Orchid Conservation & Research Center of Shenzhen, CHINA

## Abstract

A new species of Primulaceae, *Primula undulifolia*, is described from the hilly area of Hunan province in south-central China. Its morphology and distributional range suggest that it is allied to *P*. *kwangtungensis*, both adapted to subtropical climate, having contiguous distribution and similar habitat, growing on shady and moist cliffs. Petioles, scapes and pedicels of them are densely covered with rusty multicellular hairs, but the new species can be easily distinguished by its smaller flowers and narrowly oblong leaves with undulate margins. Molecular phylogenetic analysis based on four DNA markers (ITS, *mat*K, *trn*L-F and *rps*16) confirmed the new species as an independent lineage and constitutes a main clade together with *P*. *kwangtungensis*, *P*. *kweichouensis*, *P*. *wangii* and *P*. *hunanensis* of *Primula* sect. *Carolinella*.

## Introduction

The genus *Primula* L. can be explicitly recognized by its perennial habitat, basal rosette of leaves, heterostylous flowers with obvious tube and capsular fruits, with rare exemptions (with homostylous flowers, which have only one flower type, with anthers and stigma at the same level) [1, 2]. The heterostyly is a distinctive feature, with species possessing short-styled thrum-type flowers with the style included in the corolla tube, and the long-styled pin-type flowers with long style and the stigma presented at the throat of the tube. Recent molecular phylogenetic studies indicated that *Primula* (with *Dionysia* Fenzl, *Sredinskya* (Stein) Fed., *Dodecatheon* L., and *Cortusa* L. nested) is a major monophyletic clade of Primulaceae, while several genera traditionally included within the Primulaceae (e.g. *Samolus* L. and *Lysimachia* L.) have been transferred to the Theophrastaceae or to the Myrsinaceae [3–7]. This genus consists of more than 500 species, mainly distributed in the temperate zone of the Northern Hemisphere [2, 8, 9]. The modern center of diversity of *Primula* is in southwestern China (particularly in western Sichuan, eastern Xizang, and northwestern Yunnan), including approximately 300 species [1, 8].

Pax [10] proposed a preliminary infrageneric system of *Primula* and 20 sections were recognized, but the demarcation was unclear. Smith et al. [11] divided this genus into 31 sections further, and Hu [1] revised Chinese species of *Primula* and followed this treatment. To summary the relationships among the 31 sections delimited by Smith et al. [11], Wendelbo [12] assigned them into seven subgenera (i.e. subgen. *Sphondylia* (Duby) Ruprecht, subgen. *Auriculastrum* Schott, subgen. *Primula*, subgen. *Auganthus* (Link) Wendelbo, subgen. *Carolinella* (Hemsl.) Wendelbo, subgen. *Craibia* Wendelbo, and subgen. *Aleuritia* (Link) Wendelbo). In the recent system of Richards [9], another redefined seven subgenera, subgen. *Sphondylia* (Duby) Ruprecht, subgen. *Auriculastrum* Schott, subgen. *Primula*, subgen. *Auganthus* (Duby) Wendelbo, subgen. *Carolinella* (Hemsl.) Wendelbo, subgen. *Pinnatae* Richards, and subgen. *Aleuritia* (Duby) Wendelbo, were delimited.

Recently, some molecular phylogenetic analyses focusing on *Primula* have found that most of the subgenera and sections in the traditional subgeneric classification of *Primula* were non-monophyletic [5, 7, 13, 14]. Some of the diagnostic characters, such as the calyptrate capsule, might have evolved more than once in *Primula*, even in Primulaceae [14, 15]. The subgen. *Carolinella* was nested within subgen. *Auganthus* and differentiates into four separate lineages [14, 15]. However, a formal taxonomic treatment of the genus *Primula* based on the latest molecular phylogenetic studies has not yet been proposed [13]. The traditional morphological characters (including the calyptrate capsule) are still the main evidence to establish subgroups of *Primula* in floras and taxonomical studies [16].

Subgenus *Carolinella*, characterized by capsules opening by a lid (calyptrate), includes the sole sect. *Carolinella* (Hemsl.) Pax, which is a small group in *Primula*, with 12 species (*P*. *henryi* (Hemsl.) Pax, *P*. *chapaensis* Gagnep., *P*. *partschiana* Pax, *P*. *rugosa* N.P. Balakr., *P*. *kweichouensis* W.W. Smith, *P*. *kwangtungensis* W.W. Smith, *P*. *wangii* F.H. Chen & C.M. Hu, *P*. *levicalyx* C.M. Hu & Z.R. Xu, *P*. *calyptrata* X. Gong & R.C. Fang, *P*. *hunanensis* G. Hao, C.M. Hu & X.L. Yu, *P*. *intanoensis* T. Yamazaki, *P*. *cardioeides* W.W. Smith & H.R. Fletcher) endemic to southwestern China and adjacent Vietnam and Thailand [2, 9, 16–19].

At first, members of sect. *Carolinella* were described as a separate genus, named as *Carolinella* Hemsl., consisting of only three species, *Carolinella henryi* Hemsl. (≡*Primula henryi* (Hemsl.) Pax), *Carolinella cordifolia* Hemsl. (≡*Primula partschiana* Pax) and *Carolinella obovata* Hemsl. (≡*Primula rugosa* Balakr.) [20, 21]. This genus was distinguished from *Primula* by the large cordate leaves and calyptrate capsule [21]. Pax & Knuth [22] considered that the calyptrate capsules of *Carolinella* are in fact not operculate but rather dehisce by irregular calyptra as occurring in other *Primulas*, and merged it into *Primula* as a section. Subsequent taxonomists all followed this treatment [1, 2, 9, 11]. Although the sect. *Carolinella* was been recognized for a long time, some members (i.e. *P*. *kwangtungensis* and *P*. *kweichouensis*) have ever been placed in sect. *Obconicolisteri* Balf. f. [11].

In the early 2014, during field expedition in southwest Hunan, China, one of the authors fund a distinct *Primula* species with undulant margin of leaves. At first sight, this species is similar to *P*. *kwangtungensis*, and is also easily confused with some variants of *P*. *obconica* Hance (belongs to sect. *Obconicolisteri*). Further morphologic investigation of both flowering and fruiting individuals turned out that it represents an undescribed taxon. Evidenced by the unique calyptrate capsule among sections in *Primula*, it is supposedly affiliated to the sect. *Carolinella*. Thus, here we not only make a morphological comparison with related species but also conduct a phylogenetic analysis to determine the systematic position of the new species.

## Materials and Methods

### Ethics statement

All samples employed in this study are not endangered nor protected in the sampled area, and none of the sampled locations are privately owned or protected by any law. No specific permits are required for the present study.

### Nomenclature

The electronic version of this article in Portable Document Format (PDF) in a work with an ISSN or ISBN will represent a published work according to the International Code of Nomenclature for algae, fungi, and plants, and hence the new names contained in the electronic publication of a PLOS article are effectively published under that Code from the electronic edition alone, so there is no longer any need to provide printed copies.

In addition, new names contained in this work have been submitted to IPNI, from where they will be made available to the Global Names Index. The IPNI LSIDs can be resolved and the associated information viewed through any standard web browser by appending the LSID contained in this publication to the prefix http://ipni.org/. The online version of this work is archived and available from the following digital repositories: PubMed Central, LOCKSS.

### Taxon sampling

The present phylogenetic analysis was based on the framework of *Primula*, with subgen. *Auganthus* as focal group, with reference to recent molecular phylogenetic studies [4–6, 13–15]. We downloaded 311 sequences for 66 species of *Primula* from GenBank. In addition, we sequenced five species including the new species and the recently described *P*. *hunanensis*. Totally, the ingroup included 342 sequences (86 accessions) of 68 species, representing all main sections of the genus *Primula* and all nine sections of subgenus *Auganthus* established by Richards [9]. Two species of the related genera to *Primula*, i.e. *Androsace sublanata* Hand.-Mazz. and *Soldanella alpina* L., were selected as outgroups following previous phylogenetic studies [5, 6, 13, 23]. Voucher information and GenBank accession numbers are listed in S1 Table.

### DNA extraction and sequencing

Total genomic DNA was extracted from silica gel-dried plant leaves following a modified CTAB protocol of Doyle & Doyle [24]. The nucleotide sequences of the nuclear ribosomal internal transcribed spacers (ITS, including ITS1-5.8S-ITS2) and three chloroplast regions (*mat*K, *rps*16 and *trn*L-F), which were employed to infer phylogenetic relationships of *Primula* in previous studies, were analyzed in this study. For ITS, the primers, PCR amplification and sequencing conditions followed Liu et al. [14]. For chloroplast regions, the primers, PCR amplification and sequencing conditions followed Yan et al. [15], except the *mat*K region of two samples (see S1 Table for details) that followed Yan et al. [25].

### Sequence alignment and phylogenetic analysis

SeqMan (DNASTAR Inc., Madison, WI, USA) was used to assemble sequences. Multiple-sequence alignment for each marker was made using Clustal X 1.83 [26], and then minor manually adjusted using Se-Al version 2.0a11 [27]. Following the treatment of Liu et al. [14], combined phylogenetic analysis (including all the four datasets) was carried out using maximum parsimony (MP) and Bayesian inference (BI).

MP analysis was conducted using heuristic searches of random taxon addition for 100 replicates through PAUP* version 4.0b10 [28], and tree bisection–reconnection (TBR) branch swapping with no limit to the number of trees saved. All characters were weighted equally and gaps were considered as missing data. Relative support for individual clades was evaluated by Bootstrap analyses, which was conducted from 1000 replicates of heuristic search with TBR branch-swapping and saving multiple trees [29].

Bayesian analysis was carried out using MrBayes version 3.2.1 [30]. Best-fitting nucleotide substitution model for the combined data matrix was selected by ModelTest version 3.7 [31]. The Bayesian Markov Chain Monte Carlo (MCMC) algorithm was run for 2000000 generations under a GTR + I + G substitution model, with sampling of trees every 200^th^ generation. The initial 25% of sampled trees were discarded as burn-in after checking for stationarity and convergence of the chains with TRACER v1.6 [32]. The 50% majority-rule consensus tree was calculated from remaining trees with nodal support summarized as posterior probabilities (PP).

Bootstrap values ≥ 80 and Bayesian posterior probabilities ≥ 0.95 were considered as robust support.

### Morphological observations

We observed and measured both fresh and pressed specimens of the new species to obtain the accurate description. All the quantitative characters, like the diameter of corolla, which were easily changed from fresh to dry, were measured from forty fresh individuals (with half thrum flowers and half pin flowers) in the field, randomly. Other qualitative characteristics, like the texture of leaf, which were stable, were observed from ten pressed specimens. Specimens of the related species, i.e. *P*. *henryi*, *P*. *chapaensis*, *P*. *partschiana*, *P*. *rugosa*, *P*. *kweichouensis*, *P*. *kwangtungensis*, *P*. *wangii*, *P*. *hunanensis*, *P*. *levicalyx* and *P*. *calyptrate*, were checked and a morphological comparison was made with the new species. The occurrences of the main diagnostic characters (inflorescence types and calyx shape) of the members of *P*. sect. *Carolinella* were displayed on the resulting phylogenetic tree. The information of the examined specimens can be found in the S1 Text.

## Results

### Phylogenetic analysis

**Fig 1:**
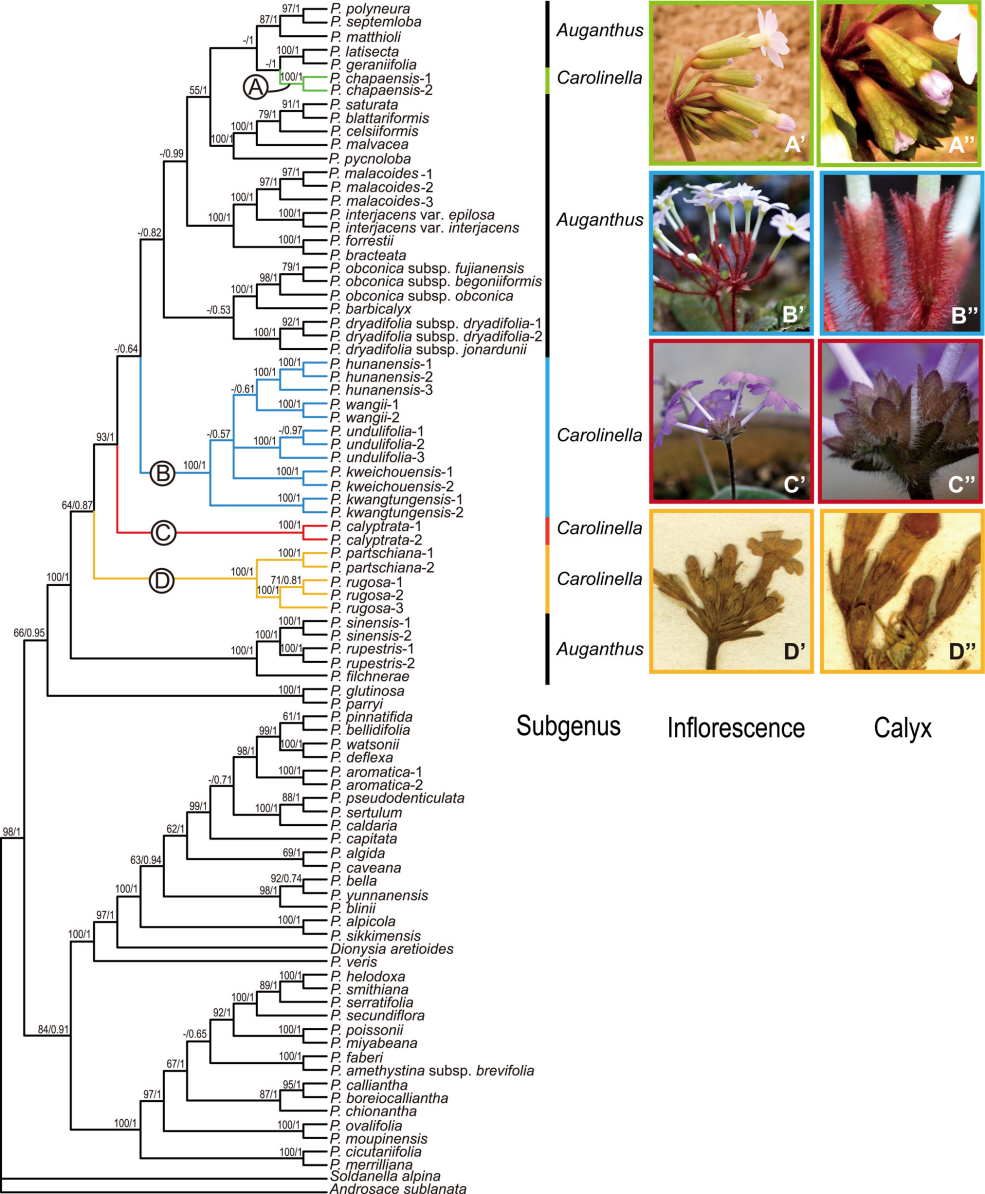
Bayesian majority consensus tree of *Primula* based on the four DNA markers (ITS, *mat*K, *trn*L-F and *rps*16) with main diagnostic characters (inflorescence types and calyx shape) mapped on. Numbers before and after the slash on the branches are parsimony bootstrap values (≥ 50%) and Bayesian posterior probabilities (≥ 0.5), respectively. Clades A-D represent the monophyletic clade of *P*. sect. *Carolinella*. (A’) Inflorescence of *P*. *chapaensis*; (B’) Inflorescence of *P*. *kwangtungensis*; (C’) Inflorescence of *P*. *calyptrate*; (D’) Inflorescence of *P*. *rugosa*; (A”) Calyx of *P*. *chapaensis*; (B”) Calyx of *P*. *kwangtungensis*; (C”) Calyx of *P*. *calyptrate*; (D”) Calyx of *P*. *rugosa*.

Aligned length of the combined dataset (ITS + *mat*K + *trn*L-F + *rps*16) was 4655 bp, of which 1901 sites were variable and 1253 were maximum parsimony informative. The majority consensus tree of the Bayesian analysis of the combined dataset is shown in Fig 1, and the topology was congruent with that of MP analysis. The combined matrix and the majority consensus tree were deposited in TreeBASE; study number S19166 (http://purl.org/phylo/treebase/phylows/study/TB2:S19166).

Species of sect. *Carolinella* were divided into four separate clades (Fig 1; clades A, B, C, D). Two samples of *P*. *chapaensis* deviated and were deeply nested within subgen. *Auganthus* (Fig 1; clade A). Other examined species of sect. *Carolinella* fell into three phylogenetically independent lineages (Fig 1; clades B, C, D). The clade including all the samples of *P*. *partischiana* and *P*. *rugosa* was first diverged from the remaining group of subgen. *Carolinella* (Fig 1; clade D). Then the clade of *P*. *calyptrata* was differentiated as an independent lineage (Fig 1; clade C). The new species together with *P*. *hunanensis*, *P*. *wangii*, *P*. *kweichouensis* and *P*. *kwangtungensis*, formed a clade and became the sister to the majority members of subgen. *Auganthus* (Fig 1; clade B). All the three samples of the new species formed a robust monophyletic group (Fig 1; clade B).

### Taxonomic treatment

**Fig 2:**
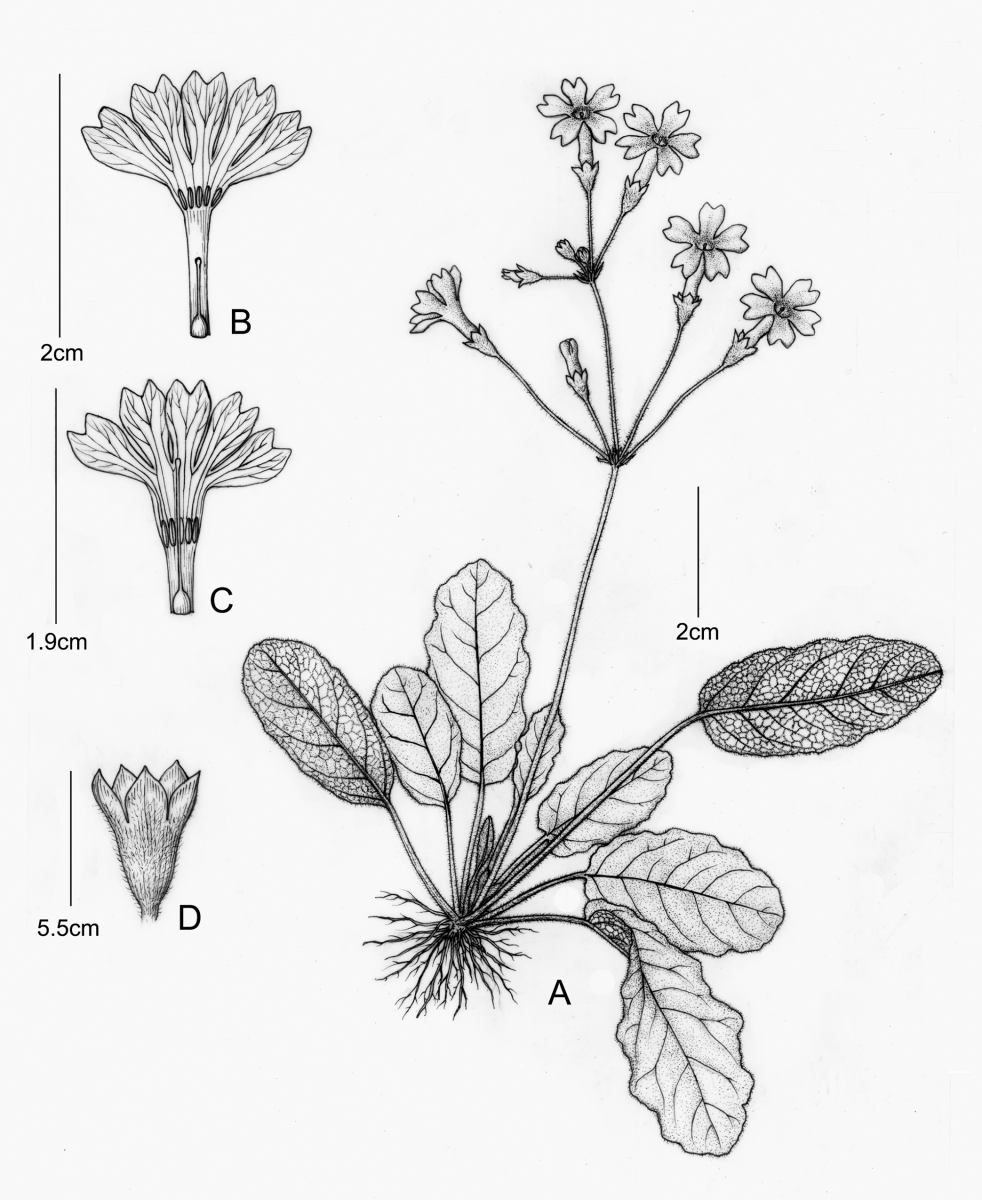
***Primula undulifolia* sp. nov.** (A) Plant; (B) Short-styled Flower; (C) Long-styled Flower; (D) Calyx. Drawn by Yunxiao LIU, from the holotype.

**Fig 3:**
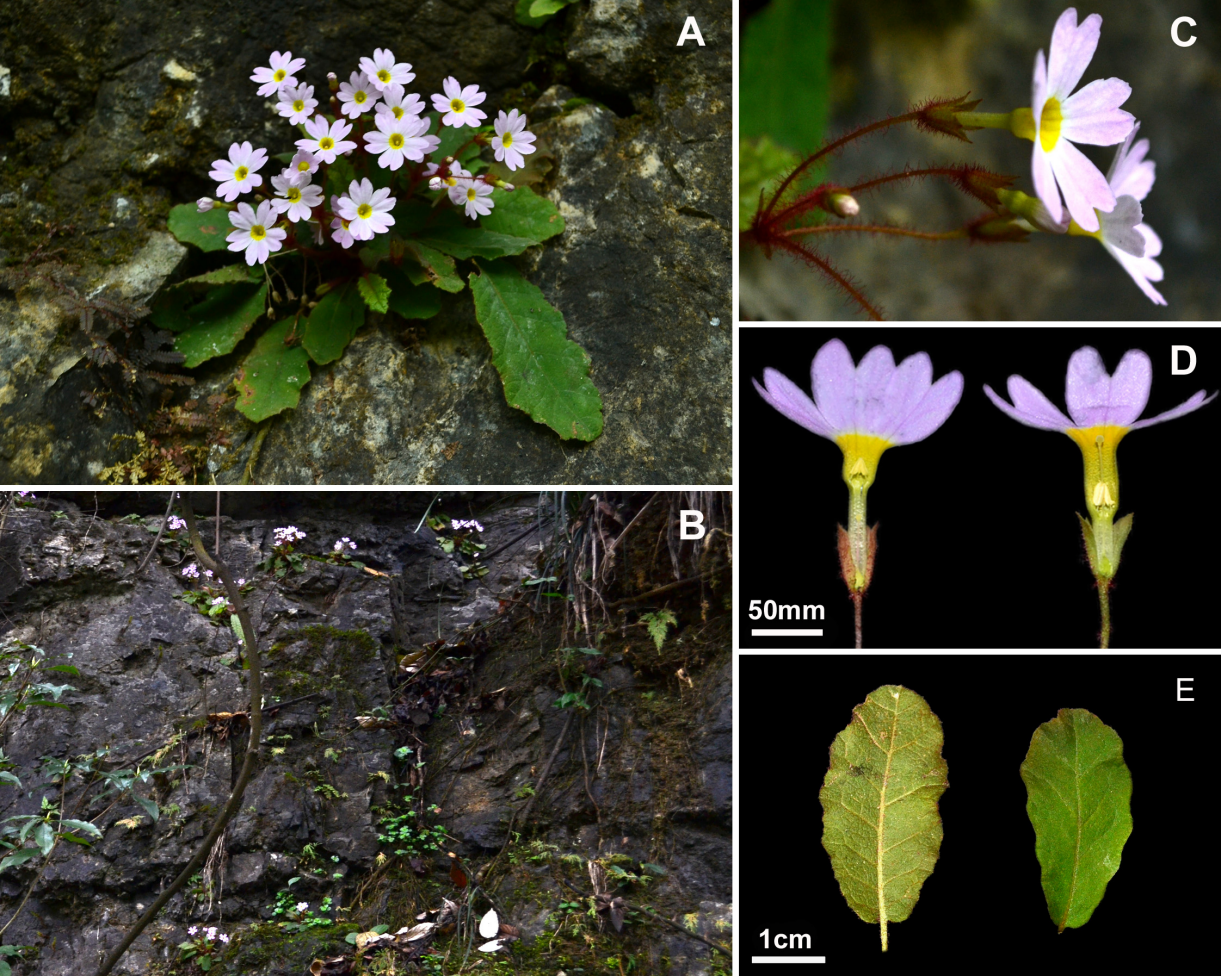
***Primula undulifolia* sp. nov.** (A) Habit in Flowering; (B) Type Locality; (C) Calyx; (D) Pin and Thrum Flowers; (E) Leaf. Photographed by Yuan XU.

***Primula undulifolia*** G. Hao, C.M. Hu & Y. Xu, sp. nov. [urn:lsid:ipni.org:names:XXXXX] (Figs 2 and 3) Type: China. Hunan province: Yongzhou City, Dong’an Xian, Damiaokou town, Xiexi village, 348 m a.s.l., 4 Mar., 2015, *Y*. *Xu & T*. *J*. *Liu*, *Xu150001* (holotype: IBSC, isotype: IBSC).

### Diagnosis

At first sight the new species seems to be some variants of *P*. *obconica* Hance, but the calyptrate capsule clearly shows that its true affinity is with the species of section *Carolinella*, and the most closely related species is *P*. *kwangtungensis*, but the new species can be easily distinguished from *P*. *kwangtungensis* by its smaller flowers and narrowly oblong leaves with undulate margins.

### Description

Herbs perennial, efarinose; rhizome very short, with numerous fibrous roots. Leaves in a rosette; petiole slender, 2.5–5.5 cm long, half the length of the blade to almost as long as it, densely covered with rusty curled multicellular hairs; leaf blade oblong to oblong-elliptic, 2.5–6.5 × 1.2–4 cm, papery when dry, abaxially sparsely pilose along main veins and veinlets, adaxially sparsely pilose along midvein, otherwise glabrous, apex rounded, base subrounded, sometimes slightly cordate and asymmetrical, margin undulate, with irregular wavy undulations, ciliate; lateral veins 5–6 on each side of midvein, c. 45° angled to midvein. Scapes 2 to many per plant, 2.5–8.5 cm long, together with pedicels densely covered with rusty multicellular hairs; umbel 3–5-flowered, solitary or with a second whorl of flower; bracts linear, 3–5 mm long, less than 1 mm broad, apex acute, more or less pilose at base; pedicels slender, 1.5–2 cm long, lengthening to 2.5 cm in fruit. Flowers distylous. Calyx campanulate to tubular-campanulate, 5–5.5 mm, split to a third of its length; tube pilose outside; lobes ovate, c. 1.5 mm wide, apex acute, glabrous. Corolla pale violet-purple, annulate, limb c. 1.5 cm wide, lobes obovate, c. 7 × 4 mm, bilobed, lobules entire; corolla tubes c. 9 mm long, thrum flowers with stamens inserted towards apex, style slightly exceeding the calyx; pin flower with stamens inserted on the middle of the corolla tube, slightly exceeding the calyx, style equaling the corolla tube. Capsule c. as long as the persistent calyx, with a fragile wall, at first opening by a thicker lid.

### Distribution, habitat and phenology

**Fig 4:**
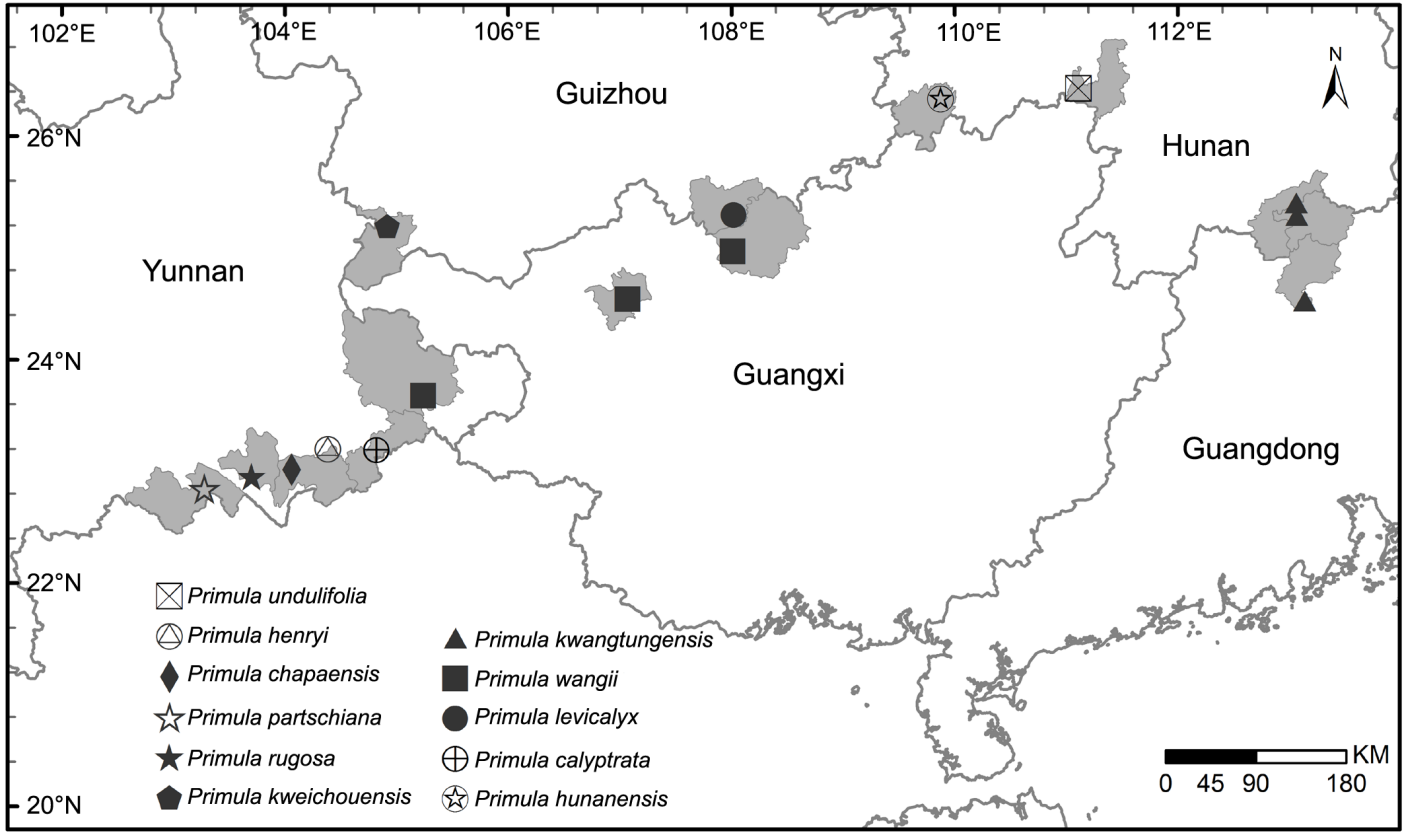
Distribution of *Primula undulifolia* sp. nov. and other Chinese species of sect. *Carolinella*.

The new species is presently known only from the type locality in Dong’an Xian of Hunan Province (Fig 4). It grows on shady and moist cliffs of karst landform, alt. 348 m. Flowering in March to April.

### Etymology

The species epithet “*undulifolia*” refers to the leaf margin of the new species with wavy and shallow undulations.

### Conservation status

The new species is presently known only from the type collection with two small populations. The habitat adjoins to the farmland, and become fragile due to deforestations and farming activities. Based on current information and according to IUCN red list criteria [33], its conservation status is evaluated as ‘Critically Endangered’ (CR) (B1abiii).

## Discussion

### Taxonomy and phylogenetic relationships

Based on the similar habit and vegetative characters, sect. *Carolinella* was considered having affinity with sect. *Obconicolisteri*, but can be distinguished by its calyptrate capsule [19]. The new species also seems as a variant of *P*. *obconica* by the leaf morphology at first sight. In this study, traits of calyptrate capsule, tubular-campanulate calyx and rusty indumentum, as well as the molecule phylogenetic result all reveal that *P*. *undulifolia* should be affiliated to sect. *Carolinella* defined by Hu [19], and are closely related to *P*. *hunanensis* and *P*. *kwangtungensis*. While species from sect. *Obconicolisteri* generally have a broadly campanulate calyx.

The non-monophyly of sect. *Carolinella* shown in this study has been verified by the recent phylogenetic analyses [14, 15]. All the examined species of sect. *Carolinella* are deeply nested within subgen. *Auganthus* and divided into four clades (Fig 1; clades A, B, C, D). We confirmed the systematic position of the new species *P*. *undulifolia*, which clusters with *P*. *hunanensis*, *P*. *wangii*, *P*. *kweichouensis* and *P*. *kwangtungensis* (Fig 1; clade B). Morphologically, this clade can be distinguished from other clades of sect. *Carolinella* (Fig 1; clades A, C and D) by the umbel with heterostylous flowers (Fig 1B’). In contrast, species of clade A and D have abbreviated raceme (Fig 1A’ and 1D’) and clade C has superimposed umbel with homostylous flowers (Fig 1C’). Additionally, calyx lobes of clade B are ovate-triangular (Fig 1B”), while that of clade A are ovate and veined (Fig 1A”), clade C are ovate (Fig 1C”), clade D are triangular and apex acuminate to caudate (Fig 1D”).

Although, the phylogenetic relationship of clade B (Fig 1) is unresolved, all the three accessions of the new species *P*. *undulifolia* constitute a robust monophyletic group. Morphologically, *P kwangtungensis* is closely related to *P*. *undulifolia* with the same traits of indumentum and calyx, and it is sister to all the other species (including *P hunanensis*, *P*. *wangii*, *P kweichouensis* and *P*. *undulifolia*) of clade B (Fig 1). Meanwhile, except for *P kwangtungensis*, the remaining species of clade B (Fig 1) are distinct for each other: pedicel and calyx of *P hunanensis* are glabrous, leaf blade of *P wangii* is more or less cordate at base, calyx of *P*. *kweichouensis* split to 2/3 of its length and leaf margin of *P*. *undulifolia* is undulate. Otherwise, the geographical distributions of all five species, included in clad B (Fig 1), are non-overlapping (Fig 4).

### Diversification of *Primula* in subtropical mountains

Previous studies [2, 8, 16, 25] indicated that the geographical distribution of the genus *Primula* is an outstanding example of high diversity and endemism. As a whole the distribution of the genus is worldwide, but with most of the species concentrated in a narrow area, stretching from East Himalaya to the high mountains of western provinces Yunnan and Sichuan of China. Of the 300 species in China, more than 80% occur in this area.

Meanwhile the recognized clades comprising the new species (Fig 1; clades A-D) in the present study represents a few outliers that are found in the subtropical mountains, with a distributional range from SE Yunnan and W Guangxi along Nanling mountain extending to N Guangdong, in a narrow (c. 250 km wide) strip of area, between latitude 23°N to 26°N, longitude 103°E to 113°E (Fig 4). This region harbors almost all the species of sect. *Caronlinella* (except *P*. *intanoensis* distributed in Thailand and *P*. *cardioeides* distributed in Vietnam) and a few members of sect. *Monocarpisae* (i.e. *P*. *malacoides* Franch. and *P*. *cavaleriei* Petitm.) and sect. *Obconicolisteri* (i.e. *P*. *barbicalyx* Wright, *P*. *obconica* Hance and *P*. *apicicallosa* D. Fang). Compared with the alpine species, the subtropical species of *Primula* have wide blade and conspicuous petiole. These species also demonstrated highly endemism and abundant diversity in the special ecological niche of Danxia landform and karst landform of this stripe. The morphological and distributional range of the new species suggest that it is closely related to *P*. *hunanensis* and *P*. *kwangtungensis*, all adapted to subtropical climate, having adjacent ranges and similar habitat, growing on acidic soil of Danxia landform or karst landform.

## Supporting Information

S1 Table Species, voucher information and GenBank accession numbers. (XLSX)

S1 Text The examined specimens of species related to the new taxon. (DOCX)
