# Correction: Morphological and Molecular Phylogenetic Data Reveal a New Species of *Primula* (Primulaceae) from Hunan, China

**DOI:** 10.1371/journal.pone.0165355

**Published:** 2016-10-20

**Authors:** The PLOS ONE Staff

The appropriate GUID numbers are missing from the “Taxonomic treatment” subsection of the Results. The publisher apologizes for the error. The correct sentence should read as: **Primula undulifolia** G. Hao, C.M. Hu & Y. Xu, sp. nov. [urn:lsid:ipni.org:names: 60472693–2] (Figs 2 and 3) Type: China. Hunan province: Yongzhou City, Dong’an Xian, Damiaokou town, Xiexi village, 348 m a.s.l., 4 Mar., 2015, *Y*. *Xu & T*. *J*. *Liu*, *Xu150001* (holotype: IBSC, isotype: IBSC).

There is an error in both Fig 2 and Fig 3. The scale bar is incorrect. Please see the corrected figures here.

**Fig 2:**
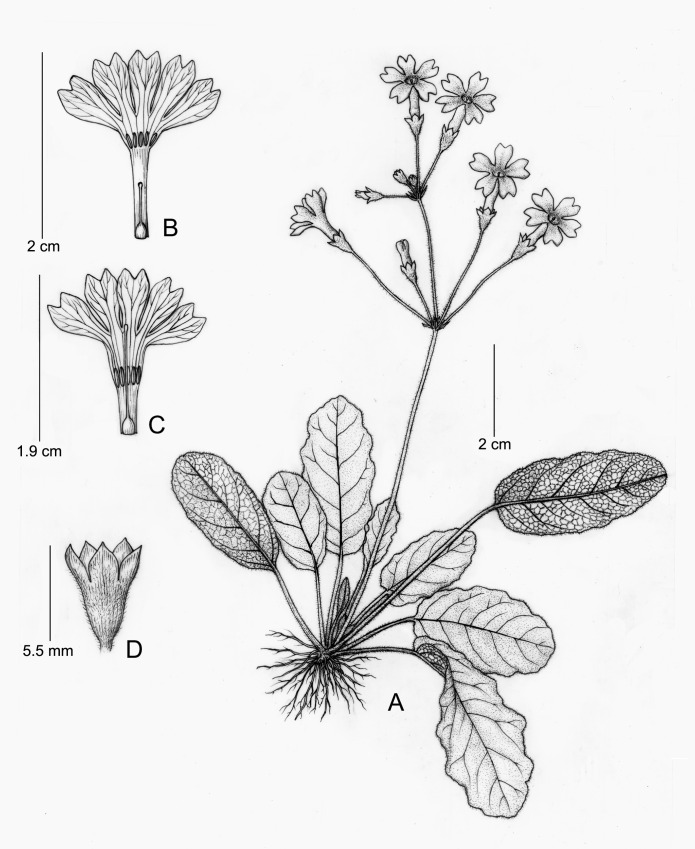
**Primula undulifolia sp. nov.** (A) Plant; (B) Short-styled Flower; (C) Long-styled Flower; (D) Calyx. Drawn by Yunxiao LIU, from the holotype

**Fig 3:**
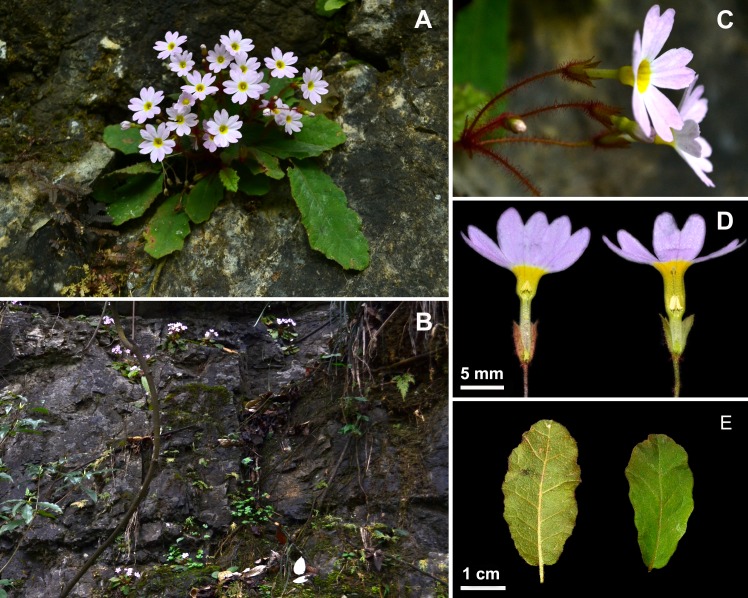
**Primula undulifolia sp. nov.** (A) Habit in Flowering; (B) Type Locality; (C) Calyx; (D) Pin and Thrum Flowers; (E) Leaf. Photographed by Yuan XU.

## References

[1] Xu Y, Yu X-L, Hu C-M, Hao G (2016) Morphological and Molecular Phylogenetic Data Reveal a New Species of Primula (Primulaceae) from Hunan, China. PLoS ONE 11(8): e0161172 doi:10.1371/journal.pone.0161172 27579832 10.1371/journal.pone.0161172 PMC5007043

