# Supplementary material for: Morphological and Molecular Phylogenetic Data Reveal a New Species of Primula (Primulaceae) from Hunan, China
Source: PLoS One. 2016 Aug 31;11(8):e0161172. doi: 10.1371/journal.pone.0161172 (PMC5007043; doi:10.1371/journal.pone.0161172)
Supplement: S1 Text — (DOCX) [file pone.0161172.s002.docx]

S1 Text. The examined specimens of species related to the new taxon.

*Specimens of Primula* *calyptrata examined*: -CHINA. Yunnan: Malipo Xian, Xiajinchang, 1870 m, 16 Apr. 2001, *X. Gong 02815* (Holotype KUN); Malipo Xian, Xiajinchang, 19 May 2001, *G. Hao 247* (IBSC); Malipo Xian, Zhongzai, 1700 m, 23 Mar. 2002, *Y. M. Shui et al. 20336* (Paratype KUN).

*Specimens of* *Primula chapaensis examined*: -CHINA. Yunan: Maguan Xian (Ma-Kwan Hsien), 1700 m, 1 Mar. 1933, *H. T. Tsai 51854* (Isotype E); Maguan Xian, 1300-1700 m, *Y. M. Shui et al. 41158* (IBSC, KUN, PE); Maguan Xian, Gulinqing, 1400-1800 m, 15 Nov. 1985, *T. R. Xu 5642* (IBSC).

*Specimens of* *Primula henryi examined*: -CHINA. Malipo Xian, Babu Qu, 1620 m, 14 May 1965, *S. Z. Wang 309* (KUN); Mengzi, *A. Henry 10735* (Type K).

*Specimens of Primula hunanensis examined*: -CHINA. Hunan: Tongdao xian, Lingkou Zhen, Taipingyan Cun, 486 m, 4 Mar. 2014, *Y. Xu 140021* (Holotype IBSC); Tongdao xian, Lingkou Zhen, Taipingyan Cun, 25 Apr. 2013, *Y. Xu & T. J. Liu 130015* (Paratype IBSC); Tongdao xian, Lingkou Zhen, Taipingyan Cun, 22 Jul. 2013, *Y. Xu 130197* (Paratype IBSC).

*Specimens of Primula kwangtungsis examined*: -CHINA. Guangdong: Lechang Xian, Pingshi, 170 m, 26 Feb. 1942, *S. Q. Chen 220* (IBSC); Ruyuan Xian, Dajiaoling, 28 Mar. 1934, *X. P. Gao 53981* (Isosyntype IBSC); Ruyuan Xian, Lankeng, 6 Oct. 1933, *X. P. Gao 53341* (Isosyntype IBSC). Hunan: Yizhang Xian, Baishidu Zhen, Xinche cun, 336 m, 9 Mar. 2013, *Y. Xu & X. Wu 130003* (IBSC).

*Specimens of* *Primula kweichouensis examined*: -CHINA. Guizhou (Kweichou): Houang-ts’ao-pa, Jan. 1919, *J. Cavalerie 4602* (Syntype E, K, P); Xingyi Xian, Malinghe, 1019 m, 16 Mar. 2013, *M. Tang & Y. Zhang ZY80* (IBSC).

*Specimens of Primula* *levicalyx examined*: -CHINA. Guizhou: Libo Xian, 900 m, 5 Apr. 1984, *Z. R. Xu et al. L1618* (Holotype IBSC).

*Specimens of Primula partschiana examined*: -CHINA. Yunan: Mengting-Berge, 2500 m, *A. Henry 10890* (Type E); Jinping Xian, May 2002, *X. Gong s.n.* (IBSC); Jinping Xian, 26 May 2000, *N. H. Xia Feb-00* (IBSC); Jinping Xian, Yongping, 2900 m, 20 Mar. 1962, *S. K. Wu 3860* (KUN).

*Specimens of* *Primula rugosa examined*: -CHINA. Yunan: Mengtze, SW Mt. forests, *A. Henry 10626A* (Syntype E); Pingbian Xian, Mt. Dawei, May 2002, X. Gong s.n. (IBSC); Pingbian Xian, Mt. Dawei, 6 Mar. 1940, *X. Wang et al. 100237* (IBSC); Pingbian Xian, Maweichong Xiang, 1850 m, 3 Apr. 1954, *P. Y. Mao 03673* (IBSC, KUN).

*Specimens of Primula wangii examined*: -CHINA. Guangxi: Fengshan Xian, Yunfeng Dong, 13 Jul. 1928, *L. Q. Chen 92269* (IBSC); Huanjiang Xian, 650 m, 20 Apr. 1981, *W. L. Sha et al. 56659* (GXMI). Yunan: Guangnan Xian, Yanzi Dong, 7 Mar. 1940, *C. W. Wang & Y. Liu 87568* (Holotype IBSC, Isotype KUN); Guangnan Xian, Heizhiguo Xiang, Yanzidong Cun, 1700 m, 2 Apr. 2007, *G. Hao 666* (IBSC).
